# Laparoscopic Resection of a Large Symptomatic Splenic Cyst: A Case Report

**DOI:** 10.7759/cureus.54580

**Published:** 2024-02-20

**Authors:** Zuhair Ahmed, Thikra Alblowi

**Affiliations:** 1 Surgery, Prince Mohammed Bin Abdulaziz Hospital, Madinah, SAU

**Keywords:** computed tomography, laparoscopic resection, benign cyst, minimally invasive surgery, primary splenic cyst

## Abstract

Splenic cysts are a rare clinical finding, often discovered incidentally during imaging for unrelated conditions. These cysts can be congenital or acquired and may present with symptoms such as vague abdominal pain. This case report describes a 25-year-old female with no significant personal medical history but a family history indicative of a predisposition to lymphoma. She presented with epigastric pain, nausea, and anorexia. Upon examination, a palpable mass was detected in the left upper quadrant. Advanced diagnostic imaging, including computed tomography and magnetic resonance imaging, identified a large benign cystic lesion at the splenic hilum. The patient underwent laparoscopic removal of the cyst, and histopathology confirmed it to be a benign epithelial splenic cyst. The patient experienced an unremarkable postoperative recovery and significant relief from symptoms. This case highlights the importance of advanced imaging in accurately identifying and managing splenic cysts and demonstrates the effectiveness of minimally invasive surgery for such conditions.

## Introduction

Splenic cysts are a relatively uncommon clinical entity, encompassing a wide range of causes. These causes vary from congenital origins, such as epidermoid and dermoid cysts, to acquired conditions, such as post-traumatic pseudocysts and cysts that develop from infections or infarctions [1,2]. The prevalence of splenic cysts in the general population is low, and they are often discovered incidentally during imaging studies for unrelated issues [1]. However, when symptomatic, patients may present with vague abdominal discomfort, pain, or fullness, particularly as cysts grow in size and exert pressure on adjacent organs [2].

The diagnostic approach to splenic cysts has evolved significantly with advances in imaging technology. Computed tomography and magnetic resonance imaging play pivotal roles in the characterization of these lesions, aiding in the differentiation between cystic and solid splenic masses, as well as between benign and malignant potential [1,3]. Management strategies for splenic cysts depend on the cyst’s size, symptoms, and suspected cause. These strategies can range from conservative observation for small, asymptomatic cysts to surgical intervention for larger, symptomatic, or complicated cysts [1]. Laparoscopic techniques have become the preferred surgical approach, offering advantages such as reduced postoperative pain, shorter hospital stays, and quicker recovery compared to open surgery [2,3].

## Case presentation

A 25-year-old woman with no significant previous medical history presented to the clinic with a two-month history of epigastric pain. The pain was described as intermittent, not related to meals, non-radiating, and somewhat alleviated by analgesics. Accompanying symptoms included episodes of vomiting and a notably decreased appetite. The patient had no history of surgeries and no known drug allergies. A positive family history of lymphoma was reported, which raised concerns regarding potential hematologic or oncologic underpinnings for her symptoms.

During the physical examination, the patient was found to be in no acute distress, with a soft and lax abdomen. A palpable mass was identified in the left upper quadrant, which warranted further investigation to determine its nature and potential impact on surrounding structures. Initial laboratory investigations, including complete blood count, liver function tests, and renal function tests, were within normal limits.

**Figure 1:**
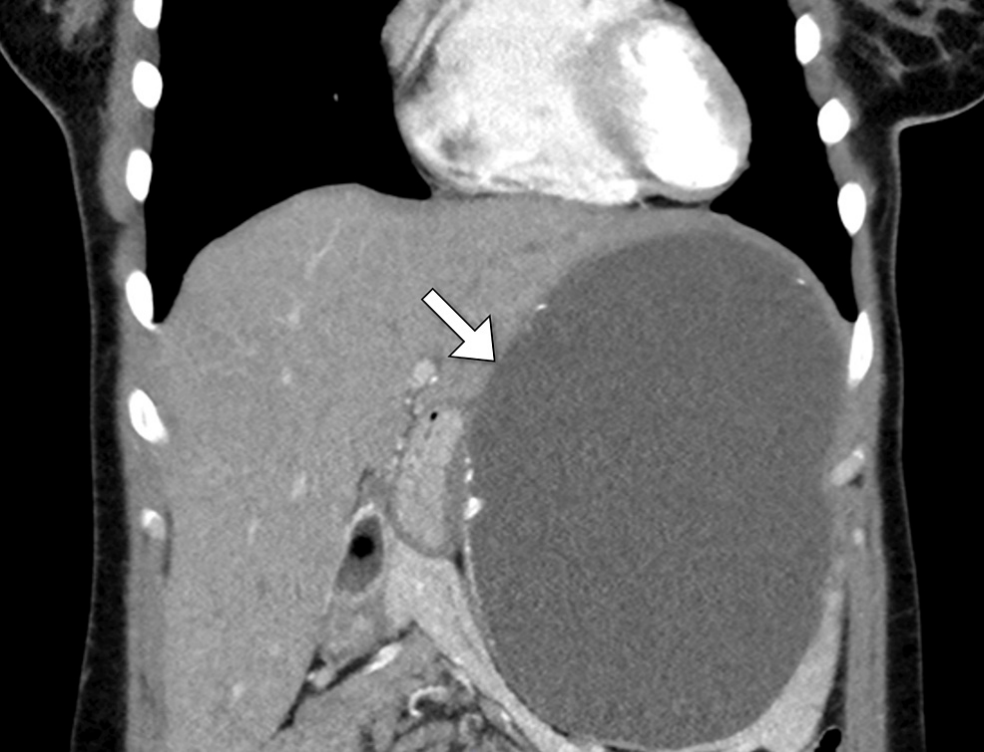
Coronal CT image of the abdomen showing a cystic lesion (arrow) within the spleen with thin marginal calcification. CT: computed tomography

**Figure 2:**
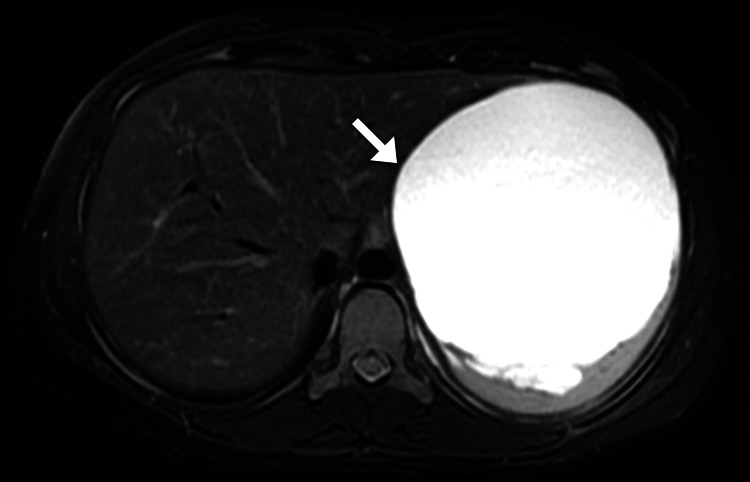
Axial T2-weighted MRI of the abdomen, displaying a lesion with high signal intensity (arrow) within the spleen. MRI: magnetic resonance imaging

Given the clinical presentation and the need for a detailed assessment of the abdominal mass, a computed tomography scan was performed as the initial step in the radiologic evaluation. The computed tomography scan revealed a large left upper abdominal quadrant cystic lesion located at the splenic hilum, anchoring into the splenic parenchyma. This mass was responsible for displacing the stomach to the right and the pancreas inferiorly, although it preserved separating fat planes between the mass, the transverse colon, and bowel loops. Notably, the splenic artery and vein remained patent. The cyst itself featured a thin wall with curvilinear focal marginal calcification and was filled with clear fluid, with no enhancing solid components observed. The imaging characteristics of the cyst suggested a benign nature (Figure 1). In light of these findings, and to better understand the cyst’s composition and precise impact on the patient’s anatomy, magnetic resonance imaging was also conducted. The scan corroborated the computed tomography findings, providing additional detail that was invaluable for surgical planning (Figure 2).

The multidisciplinary team, considering the patient’s symptoms, the size of the cyst, and its anatomical implications, recommended a laparoscopic surgical resection of the splenic cyst. The surgery involved careful exploration and needle aspiration of the cyst, during which approximately 1,700 mL of yellowish fluid was extracted. This fluid was sent for cytological analysis, which showed no malignant cells, thus ruling out cancer. Following aspiration, the cyst was meticulously resected, and the cyst wall was removed through the umbilical port, highlighting the advantages of minimally invasive surgical techniques. The patient’s postoperative course was smooth, with a marked improvement in her symptoms. Histopathological examination of the cyst confirmed it to be a benign epithelial splenic cyst, with no evidence of malignancy. The patient was scheduled for regular follow-up appointments to monitor her recovery and ensure no recurrence of the cyst, with the first follow-up at six weeks post-surgery showing excellent recovery without complications.

## Discussion

The decision to employ computed tomography for initial diagnostic imaging, followed by magnetic resonance imaging, is reflective of a strategic approach tailored to acquire comprehensive anatomical details essential for surgical planning [2,3]. Computed tomography and magnetic resonance imaging are invaluable in differentiating between cystic and solid lesions, assessing lesion complexity, and planning the surgical approach [1-3]. This meticulous imaging strategy underscores the evolving diagnostic paradigm, where precision in characterizing splenic lesions is paramount, particularly in young patients where the preservation of splenic function is a significant consideration [2,4].

Laparoscopic surgery represents a cornerstone in the management of symptomatic splenic cysts, advocated by the minimally invasive nature of the procedure and its associated benefits [1-3]. These include reduced postoperative pain, reduced risk of infection, quicker recovery times, and the preservation of splenic tissue [1,3]. The successful laparoscopic resection in this case reaffirms the procedure’s efficacy and safety, even in the management of large cysts [4]. This aligns with a growing body of literature advocating for laparoscopy as the standard care for splenic cysts, barring contraindications such as cyst location or size that preclude safe laparoscopic access.

The management strategy was further complicated by the patient’s positive family history of lymphoma, raising initial concerns regarding the cyst’s etiology [1,2]. Although the histopathological analysis revealed a benign epithelial cyst, this familial aspect necessitated a thorough diagnostic workup and careful postoperative surveillance [2-5]. This aspect of care emphasizes the importance of comprehensive history-taking and individualized management plans that consider both the immediate clinical presentation and the broader familial health context.

In the domain of splenic cyst management, this case contributes to the discourse on several fronts. It highlights the importance of an integrated diagnostic approach that leverages advanced imaging techniques for precise surgical planning. It also reaffirms the role of laparoscopic surgery as a preferred intervention for symptomatic splenic cysts, given its favorable outcomes and alignment with the principles of minimally invasive surgery [6-8]. Furthermore, the case underscores the necessity of a nuanced clinical evaluation that accounts for familial medical histories, particularly in conditions with a potential malignancy risk.

## Conclusions

The successful management of a large symptomatic splenic cyst through laparoscopic resection in a young woman highlights the critical role of precise diagnostic imaging, meticulous surgical technique, and personalized care in the treatment of splenic lesions. This case reinforces the efficacy and safety of laparoscopic approaches in preserving splenic function while addressing symptomatic cysts, emphasizing the importance of a tailored, patient-centric approach. It also underscores the necessity for ongoing research and clinical vigilance to further refine diagnostic and treatment modalities for splenic cysts, ensuring optimal outcomes in the context of individual patient histories and broader health considerations.
